# Research progress on the therapeutic effects of effective components of traditional Chinese medicine in the treatment of gastric cancer precursors through modulation of multiple signaling pathways

**DOI:** 10.3389/fonc.2025.1555274

**Published:** 2025-05-19

**Authors:** Lichao Han, Yijia Ma, Weidong Wu, Qianyue Ni, Jingri Xie, Yan Huang, Qiuyu Jin, Lili Wu, Yang Zhang

**Affiliations:** ^1^ Department of Internal Medicine, Heilongjiang University of Chinese Medicine, Harbin, China; ^2^ Acupuncture and Massage, Heilongjiang University of Chinese Medicine, Harbin, China; ^3^ Basic Theory of Chinese Medicine, Heilongjiang University of Chinese Medicine, Harbin, China; ^4^ Surgery of Chinese Medicine, Heilongjiang University of Chinese Medicine, Harbin, China

**Keywords:** gastric precancerous lesions, signaling pathway, traditional Chinese medicine, active ingredients, research progress

## Abstract

Precancerous lesions of the stomach (PLGC), a critical stage in gastric cancer development, have garnered significant attention for its prevention and treatment. PLGC refers to a series of pathological and histological changes preceding cancer. Due to its complex pathogenesis and multi-factor influence, no currently existing drug can effectively prevent or delay this process. Therefore, identifying a safe and effective treatment method remains an important research focus. In recent years, traditional Chinese medicine has demonstrated unique advantages and potential in PLGC treatment. Many studies have focused on the regulation of traditional Chinese medicine’s active ingredients on relevant signaling pathways. Studies show active ingredients can regulate cell proliferation, apoptosis, differentiation, autophagy, oxidative stress, and inflammatory response via multi-target, multi-pathway intervention of key signaling pathways like hypoxia-inducible factor 1 (HIF-1α), nuclear factor kappa-B (NF-κB), wingless-type MMTV integration site family/beta-catenin (Wnt/β-catenin), phosphoinositide 3-kinases/Protein kinase B (PI3K/AKT), Sonic Hedgehog (Shh), Janus kinase/signal transducer and activator of transcription (JAK/STAT), mitogen-activated protein kinase (MAPK), nuclear factor erythroid 2-related factor 2 (Nrf2), and more, thereby delaying or reversing PLGC progression. This paper summarizes and sorts research on the regulation of these pathways by traditional Chinese medicine’s active ingredients, seeking to provide a theoretical basis and medication reference for PLGC treatment with traditional Chinese medicine.

## 1 Introduction

Gastric cancer (GC) is one of the most common malignant tumors worldwide, with high morbidity and mortality rates, seriously endangering human life and health (1). China has a high incidence of GC, accounting for 44% of global cases, and the incidence is increasing annually (2, 3). The global burden of GC is projected to increase by 62% by 2040 (4). With the development of population aging and economy and society, GC has become one of the main causes of human death, causing a heavy economic burden on individuals and society (5). PLGC represent a multi-step carcinogenesis process, following the Correa cascade model: chronic non-atrophic gastritis → chronic atrophic gastritis (CAG) → intestinal epithelial metaplasia (IM) → dysplasia (Dys) → GC (6). Both CAG and IM are pre-cancerous states. Investigations reveal prevalence rates of CAG and IM of 16% and 13%, respectively, and may rise to 27% in high-incidence GC areas (7). Therefore, early identification and intervention of PLGC are effective measures to prevent GC development.

The clinical manifestations of PLGC mainly include stomach pain, fullness, belching, acid reflux, heartburn, loss of appetite, etc. The pathogenesis is not yet fully understood, but is thought to be related to inflammatory reactions and apoptosis (8). Western medicine believes that Helicobacter pylori (HP) infection is one of the key factors in the pathogenesis of PLGC. Persistent HP infection can induce an inflammatory reaction, causing progressive damage to the gastric mucosa, and enable the infected person to gradually develop from chronic active gastritis to chronic atrophic gastritis, and even progress to GC (9–11). Other factors such as environment, immunity, genetics, bile reflux, high salt and low fiber diet are also associated with PLGC (12, 13). For the treatment of PLGC, Western medicine mainly focuses on eradicating HP, endoscopic resection, and (cyclooxygenase-2) COX-2 inhibitor (14). Although these treatments can alleviate clinical symptoms to some extent, their overall effectiveness remains limited. For instance, Some patients develop antibiotic resistance, and after the failure of Hp eradication, alternative treatment options are limited. Even when Hp is successfully eradicated, existing gastric atrophy or intestinal metaplasia may persist. Although acid-suppressing drugs can alleviate symptoms, they do not reverse gastric mucosal atrophy or intestinal metaplasia, Prolonged use of these drugs may even exacerbate atrophy. In addition, Long-term use of COX inhibitors can lead to drug tolerance and may cause a range of adverse reactions. Traditional Chinese medicine has the advantage of multiple targets and pathways in the treatment of PLGC, which can effectively improve patient symptoms and promote pathological changes in the gastric mucosa to prevent further disease progression (15, 16). Therefore, identifying more safe and effective drug target pathways is of great research significance for delaying or reversing PLGC.

In recent years, research on signal pathways in PLGC has been vigorously carried out and achieved considerable results. Deeply understanding the relationship between the signal pathway and the molecular mechanism of PLGC, and developing and applying multi-functional and multi-target therapeutic drugs are key strategies to realize early reversal of PLGC. At present, treating PLGC by regulating relevant signal pathways has become a research hotspot, but the specific pathways and action mechanisms of traditional Chinese medicine active ingredients regulating PLGC have not been clearly expounded. Therefore, this article will summarize the achievements of traditional Chinese medicine active ingredients regulating relevant signal pathways to treat PLGC (**Table 1**), in order to provide more theoretical basis and scientific basis for the clinical medication and comprehensive treatment of PLGC.

**Table 1:** Investigation of the regulation of relevant signaling pathways by effective components of traditional Chinese medicine in the treatment of gastric precancerous lesions.

Diseases	Effective ingredients	Signaling pathways	References
PLGC	Baicalein	HIF-1α	(17)
	Ginsenoside	HIF-1α	(18, 19)
	Berberine	HIF-1α	(20)
	Atractylenolide III	HIF-1α	(21)
	Curcumol	NF-κB	(22)
	Calycosin	NF-κB	(23)
	Betulinic acid	NF-κB	(24)
	Aloin	NF-κB	(25)
	Capsaicin	NF-κB	(26)
	Artemisinin	Wnt/β-catenin	(27)
	Codonopsis polysaccharide	Wnt/β-catenin	(28)
	Gallic acid	Wnt/β-catenin	(29)
	Wogonin	Wnt/β-catenin	(30)
	Dendrobium officinale Polysaccharide	Wnt/β-catenin	(31)
	Resveratrol	PI3K/AKT	(32)
	Codonopsis oligosaccharides	PI3K/AKT	(33
	Ginsenoside Rg3	PI3K/AKT	(34)
	Epigallocatechin gallate	PI3K/AKT	(35)
	Erianin	PI3K/AKT	(36)
	Kaempferol	Sonic Hedgehog	(37)
	Yam extract	Sonic Hedgehog	(38)
	Astragaloside IV	Sonic Hedgehog	(39)
	Ginsenoside Rg1	Sonic Hedgehog	(39)
	Walnut polyphenol	JAK/STAT	(40)
	Quercetin	JAK/STAT	(41)
	Berberine	JAK/STAT	(42)
	Tanshinone IIA	JAK/STAT	(43)
	Rhein	MAPK	(44)
	Evodiamine	MAPK	(45)
	Quercetin	MAPK	(46)
	Palmitine	MAPK	(46)
	Costunolide	Nrf2	(47)
	Zerumbone	Nrf2	(48)
	Dendrobium Officinale Polysaccharide	Nrf2	(31)
	Resveratrol	Nrf2	(49)

## 2 HIF-1α signaling pathway

### 2.1 The structure and biological function of HIF-1αpathway

**Figure 1:**
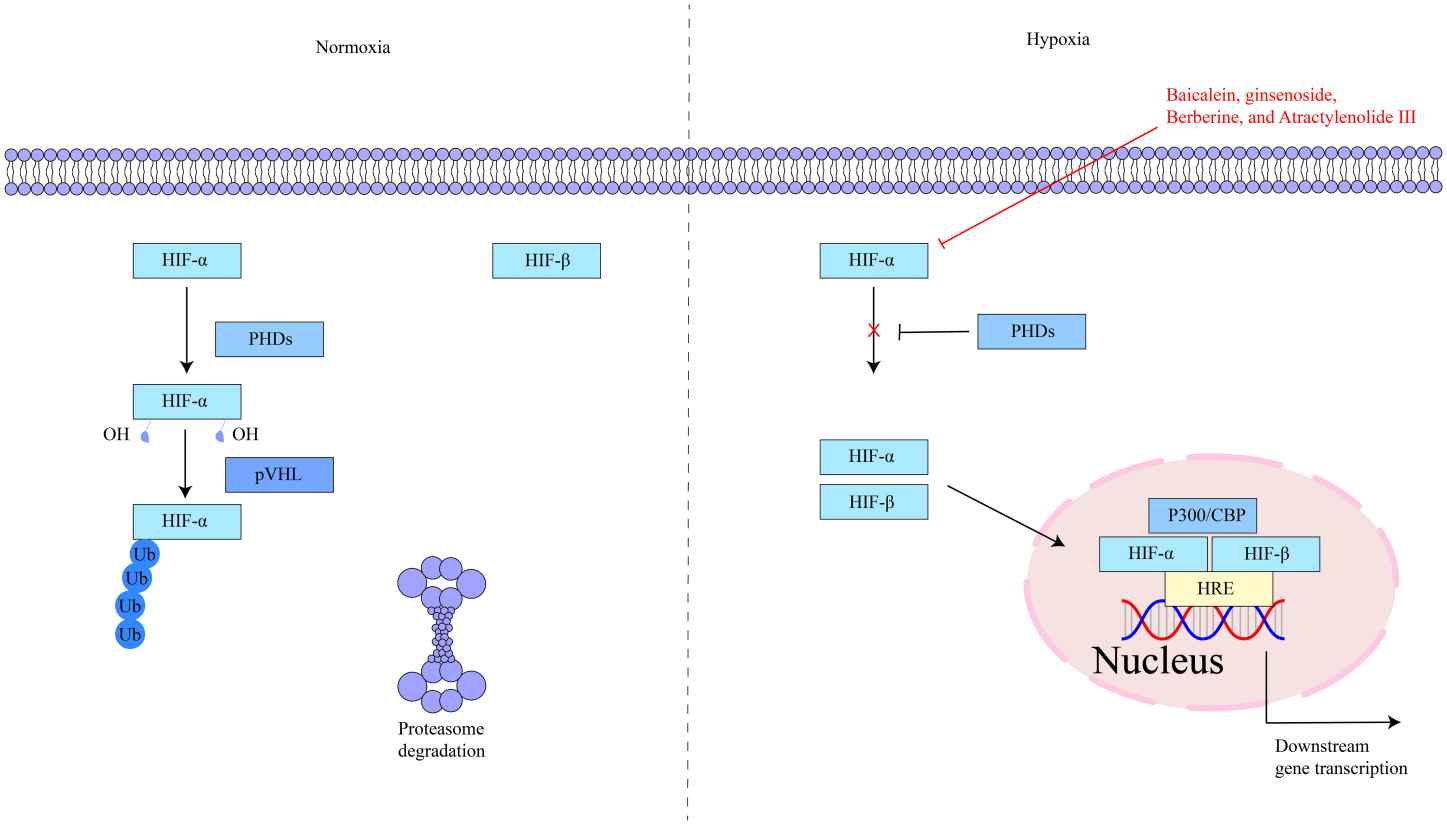
The interaction of key factors upstream and downstream in the HIF-1α signaling pathway.

HIF-1α is located on human chromosome 14q21–24 and is composed of 826 amino acids (50). Under normoxic conditions, HIF-1α is rapidly degraded, while under hypoxic conditions, it is stabilized and accumulates (51, 52). HIF-1α contains two transactivation domains (TAD), TAD-C and TAD-N. TAD-C can bind to p300/CBP, etc., to enhance the transcriptional activity of the HIF-1α protein; TAD-N can overlap with the oxygen-dependent degradation domain to enhance the stability of the HIF-1α protein (53). HIF-1α is a key regulator of the hypoxic response and has an important role in cells. Under hypoxic stimulation and factor induction, it activates the transcription of multiple genes, including glycolytic enzymes, erythropoietin, vascular endothelial growth factor, glucose transporter, etc. (54, 55). Studies have shown that hypoxia exists in inflammatory lesions and activating HIF-1α can inhibit intestinal inflammation. Simultaneously, HIF-1α is an effective protective factor that maintains mucosal barrier integrity (56). The key targets in the HIF-1α signaling pathway, as well as its upstream and downstream relationships, are depicted in **Figure 1**.

### 2.2 HIF-1α pathway and PLGC

Hypoxia is a key factor in inducing tumor metastasis. HIF-1α shows high expression in many malignant tumor cells, and even has expression in some precancerous lesion tissues. Studies have found that the expression level of HIF-1α is positively correlated with tumor invasion depth, size, degree of differentiation, and cell invasion and metastasis of GC (57, 58). During PLGC progression, the metabolic profile of cells shifts to support rapid proliferation. HIF-1α regulates the expression of genes such as glucose transporters, enhancing glucose uptake and utilization, promoting glycolysis, and providing additional energy to the diseased cells, thereby supporting their survival and proliferation (59, 60). HIF-1α is considered a common transcription factor for hypoxic cells in the tumor microenvironment, activating transcription of downstream genes including (Vascular Endothelial Growth Factor) VEGF to provide oxygen and nutrients for PLGC cells, participating in PLGC occurrence (61, 62). HIF-1α is an important oncogene. High HIF-1α levels may reflect abnormal proliferation and malignancy of GC tissues. Abnormal proliferation and malignancy of tumor tissues during GC development increase HIF-1α, raising the risk of early GC occurrence (63, 64). Epithelial-mesenchymal transition (EMT) plays a critical role in the progression of PLGC to GC. The HIF-1α pathway induces EMT in gastric epithelial cells by regulating transcription factors such as Snail and Twist, leading to the loss of epithelial polarity and intercellular connections, acquisition of mesenchymal characteristics, enhanced cell migration and invasion, and facilitating the malignant transformation of PLGC (65).

### 2.3 Effective components of traditional Chinese medicine regulate HIF-1α signaling pathway in the treatment of PLGC

The mechanism of action of active ingredients in traditional Chinese medicine for PLGC is more specific and definite, and is one important treatment method. Some active ingredients can treat PLGC by regulating the HIF-1α signaling pathway. Baicalein is mainly extracted from the dried root of Scutellaria baicalensis,with anti-inflammatory, antioxidant, anti-apoptotic, and anti-tumor functions. A study demonstrated that multiple doses of baicalein tablets, within the study’s specified dose range, were safe and well-tolerated in healthy Chinese subjects, with no severe adverse reactions. This finding provides valuable insights and a scientific foundation for the clinical application of baicalein (66). Chen et al. (17) demonstrated that Baicalein inhibits hypoxia-induced AKT phosphorylation via PTEN accumulation and reduces HIF-1α in PLGC cells, suppressing glycolysis and inhibiting cell proliferation and invasion, demonstrating therapeutic potential against PLGC. Ginsenoside is mainly extracted from Araliaceae ginseng plants, with anti-tumor and immune-enhancing functions, has been found to inhibit excessive HIF-1α and VEGF expression and down-regulate proteins such as Heat Shock Factor protein 1, Glucose Transporter 1, and Glucose Transporter 4, inhibiting early angiogenesis, improving microvessel morphology, and alleviating hypoxia tolerance in gastric mucosa, effectively shortening PLGC range (18, 19). Berberine is mainly extracted from Coptidis Rhizoma, Phellodendri Chinensis Cortex, Three Needles and other plants, with antibacterial and anti-tumor functions. Ye X et al. (20) found that Berberine inhibits the development of PLGC into malignant tumors by suppressing the HIF-1α signaling pathway, reducing the transcription and expression of genes such as VEGF, thus preventing tumor angiogenesis and disrupting the nutrient supply necessary for tumor growth and metastasis. Atractylenolide III is mainly extracted from the rhizome of Atractylodes macrocephala, with anti-inflammatory, anti-tumor, and gastrointestinal regulatory functions. Gao Y et al. (21) found through research that Atractylenolide III blocks abnormal activation of the HIF-1α and VEGF signaling pathways in PLGC, reducing high expression of HIF-1α and VEGF proteins, and down-regulating Delta - Like Ligand 4 protein expression, improving abnormal gastric mucosal microvessel morphology and reducing neovascularization to exert therapeutic effects on PLGC.

In general, the active ingredients of traditional Chinese medicine have shown unique advantages in regulating the HIF-1α signaling pathway, especially in the treatment of PLGC, showing good application prospects. Active ingredients such as Baicalein, Ginsenoside, Berberine, and Atractylenolide III can reduce inflammation, improve the PLGC microenvironment, inhibit the proliferation and differentiation of PLGC cells, and thereby hinder the progression of PLGC by regulating the HIF-1α signaling pathway.

## 3 NF-κB signaling pathway

### 3.1 The structure and biological function of NF-κB pathway

**Figure 2:**
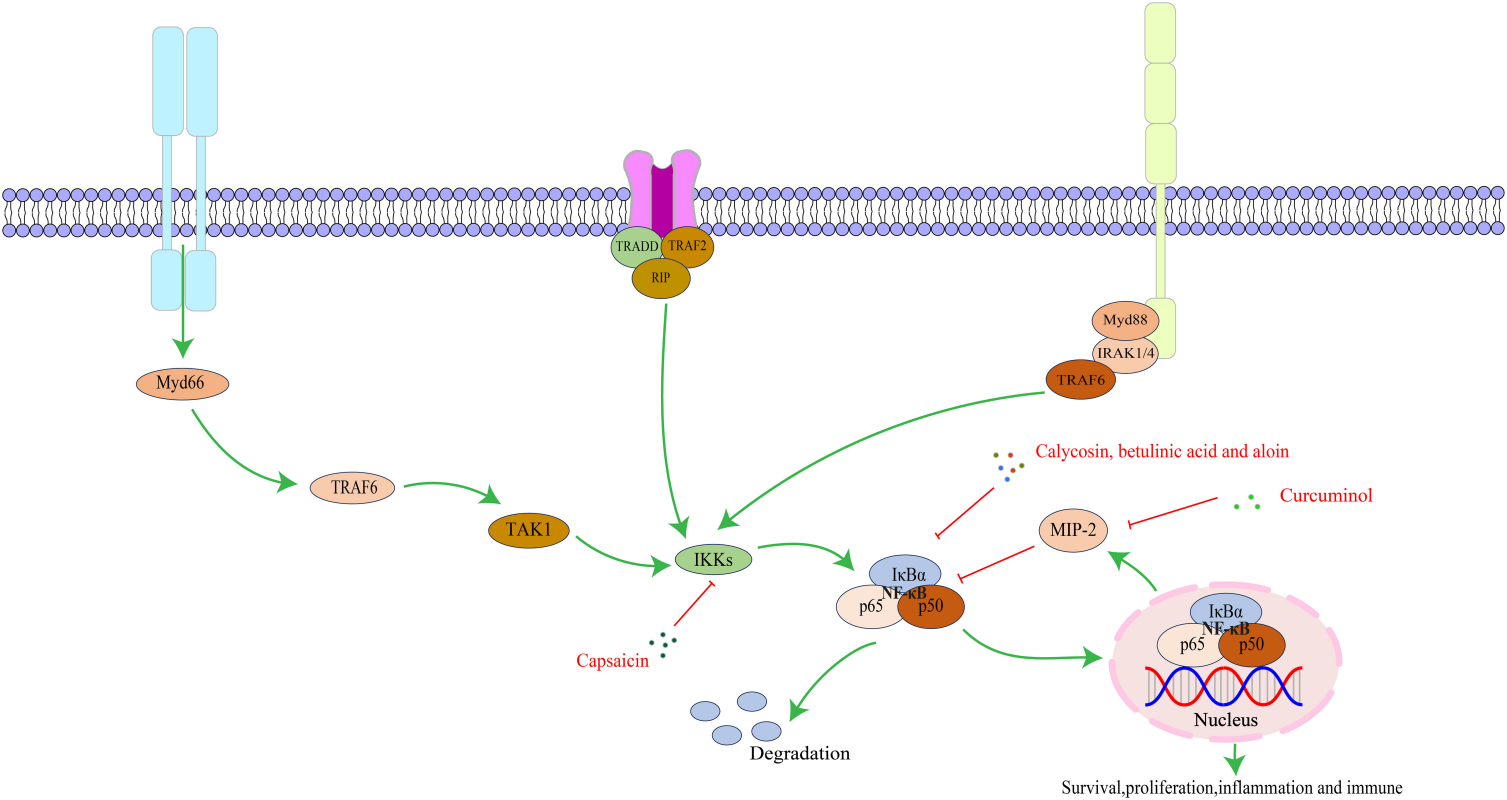
The interaction of key factors upstream and downstream in the NF-κB signaling pathway.

The NF-κB family consists of five proteins: NF-κB1 (p50/p105), NF-κB2 (p52/p100), c-Rel, RelB, and RelA/p65. Each member has a Rel homology domain-like structure region, including a dimer structure, DNA binding, and nuclear localization signal regions. Proteins form different complexes through dimerization (67, 68). The NF-κB signaling pathway is a key regulator of innate and adaptive immune responses and has functions in regulating cell proliferation, apoptosis, migration, invasion, angiogenesis, and metastasis. In several cancer animal models, abnormal NF-κB activation upregulates anti-apoptotic gene expression, causing tumor cell resistance to apoptosis and promoting growth (69). As a key mediator of the inflammatory response, NF-κB induces expression of various pro-inflammatory factors and participates in inflammasome regulation. Meanwhile, NF-κB regulates survival, activation, and differentiation of innate immune cells and inflammatory T cells (70).The key targets in the NF-κB signaling pathway, as well as its upstream and downstream relationships, are illustrated in **Figure 2**.

### 3.2 NF-κB pathway and PLGC

The NF-κB signaling pathway plays a pivotal role in the occurrence and development of GC, especially PLGC. These lesions are a key pathological stage of the transformation from normal gastric mucosa to GC, and NF-κB affects this transformation process through various mechanisms. During the progression of GC, the NF-κB signaling pathway exhibits abnormal and persistent activation, accompanied by increased expression of pro-inflammatory factors. The long-term presence of these pro-inflammatory factors can cause changes in the local microenvironment and promote malignant transformation of PLGC cells (71). NF-κB regulates the expression of cell cycle and apoptosis-related proteins. It promotes the expression of Cyclin D1, driving cells from the G1 to the S phase and accelerating proliferation. Simultaneously, NF-κB upregulates anti-apoptotic proteins such as B-cell lymphoma 2 (Bcl-2) and downregulates pro-apoptotic proteins such as Bax, inhibiting apoptosis. This imbalance between proliferation and apoptosis results in the abnormal accumulation of gastric mucosal cells, providing a foundation for PLGC development (72, 73). Liu M et al. (74) demonstrated through studies that REC8 inhibits angiogenesis by inhibiting VEGF expression mediated by the NF-κB pathway in GC cells, thereby affecting GC growth and metastasis. Inflammation is a marker of cancer and regulates cell invasion and metastasis (75). Activation of the NF-κB pathway induces the expression of various inflammatory factors, such as interleukin-6 (IL-6) and tumor necrosis factor-alpha (TNF-α), which recruit immune cells to the gastric tissue, initiating an inflammatory response. Persistent inflammation damages gastric mucosal cells and promotes abnormal cell proliferation and metaplasia, thereby increasing the risk of PLGC. (76, 77). In addition, human telomerase reverse transcriptase (hTERT) is an important marker of cell proliferation and carcinogenesis. Studies have found NF-κB activation correlates with upregulation of human telomerase reverse transcriptase (hTERT) expression. In the early stage of GC, expression of both NF-κB and hTERT is significantly upregulated, suggesting NF-κB may promote PLGC occurrence by promoting hTERT expression (78).

### 3.3 Effective components of traditional Chinese medicine regulate NF-κB signaling pathway in the treatment of PLGC

Curcumol can be extracted from turmeric, curcuma, turmeric and other plants, with anti-tumor, anti-inflammatory and other functions. It can induce apoptosis in various cancer cells by regulating key signaling pathway activity. In a randomized controlled trial, we found that curcumin is a safe and tolerable adjunct to folinic acid/5-fluorouracil/oxaliplatin chemotherapy in cancer patients, and its potential low drug resistance and multi-target anti-tumor properties provide a new idea and option for long-term treatment of tumors (79). Ma X et al. (22) confirmed that Curcumol can effectively protect gastric mucosal tissue, reduce inflammation, and inhibit the viability, invasion, and migration of GC cells by regulating the signal transduction of SDF-1α/CXCR4/NF-κB pathway, thereby treating CAG and reversing the progression of GC. Calycosin, the major active ingredient extracted from the traditional Chinese medicine Astragalus membranaceus, demonstrates anti-inflammatory, anti-tumor, and immunity-enhancing functions. Li D et al. (23) found that Calycosin can significantly reduce IM and atypical hyperplasia area in PLGC model rats, and the mechanism may be related to the regulation of p-NF-κB, NF-κB, and dopamine and cAMP-regulated phosphoprotein 32 pathway expression in PLGC mucosal tissues induced by N-methyl-N′-nitro-N- nitrosoguanidine (MNNG). Betulinic acid, extracted from birch bark and wild jujube kernel, manifests anti-inflammatory, antioxidant, and anti-tumor functions. Studies have found that Betulinic acid can down-regulate the expression of Vasodilator-stimulated phosphoprotein by inhibiting signal transduction of the NF-κB pathway, thereby inhibiting the proliferation and migration of GC cells and achieving the effect of treating PLGC (24). Aloin is mainly extracted from Aloe plants, with anti-inflammatory, antioxidant, and anti-tumor functions (80, 81). Wang Z et al. (25) discovered that Aloin suppresses the proliferation and migration of GC cells by inhibiting nicotinamide adenine dinucleotide phosphate oxidase 2-reactive oxygen species-mediated activation of the AKT-mammalian target of rapamycin (mTOR), STAT3, and NF-κB signaling pathways. Concurrently, it downregulates the expression of inflammatory factors and attenuates the inflammatory response, ultimately delaying GC progression. Pepper, known for its warming and digesting properties, contains Capsaicin as its primary herbal compound (82), which demonstrates anti-inflammatory, anti-tumor, and antioxidant functions (83). The phosphorylation of NF-κB is directly linked to inflammation and gastric injury. Saha K et al. (26) found that Capsaicin can reduce the phosphorylation of NF-κB and inhibit the expression of cytokines and NLRP3 genes via NF-κB inactivation, thereby reducing inflammatory damage to gastric tissue. Given NF-κB’s inhibitory characteristics, capsaicin can also improve the degree of mucosal inflammation and atrophy in a dose-dependent manner in CAG model rats and reduce the risk of canceration (84).

In summary, the active ingredients of traditional Chinese medicine have demonstrated good efficacy in treating PLGC by regulating the NF-κB signaling pathway. The pathogenesis of PLGC is complex, and the abnormal activation of the NF-κB signaling pathway is closely related to the occurrence and development of PLGC. Traditional Chinese medicine active ingredients such as Curcumol, Calycosin, Betulinic acid, Aloin, and Capsaicin can regulate the NF-κB signaling pathway. By inhibiting the abnormal activation of this pathway, they reduce the inflammatory response of the gastric mucosa and inhibit the proliferation and migration of PLGC cells, thereby delaying or reversing the progression of PLGC.

## 4 Wnt/β-catenin signaling pathway

### 4.1 The structure and biological function of Wnt/β-catenin pathway

**Figure 3:**
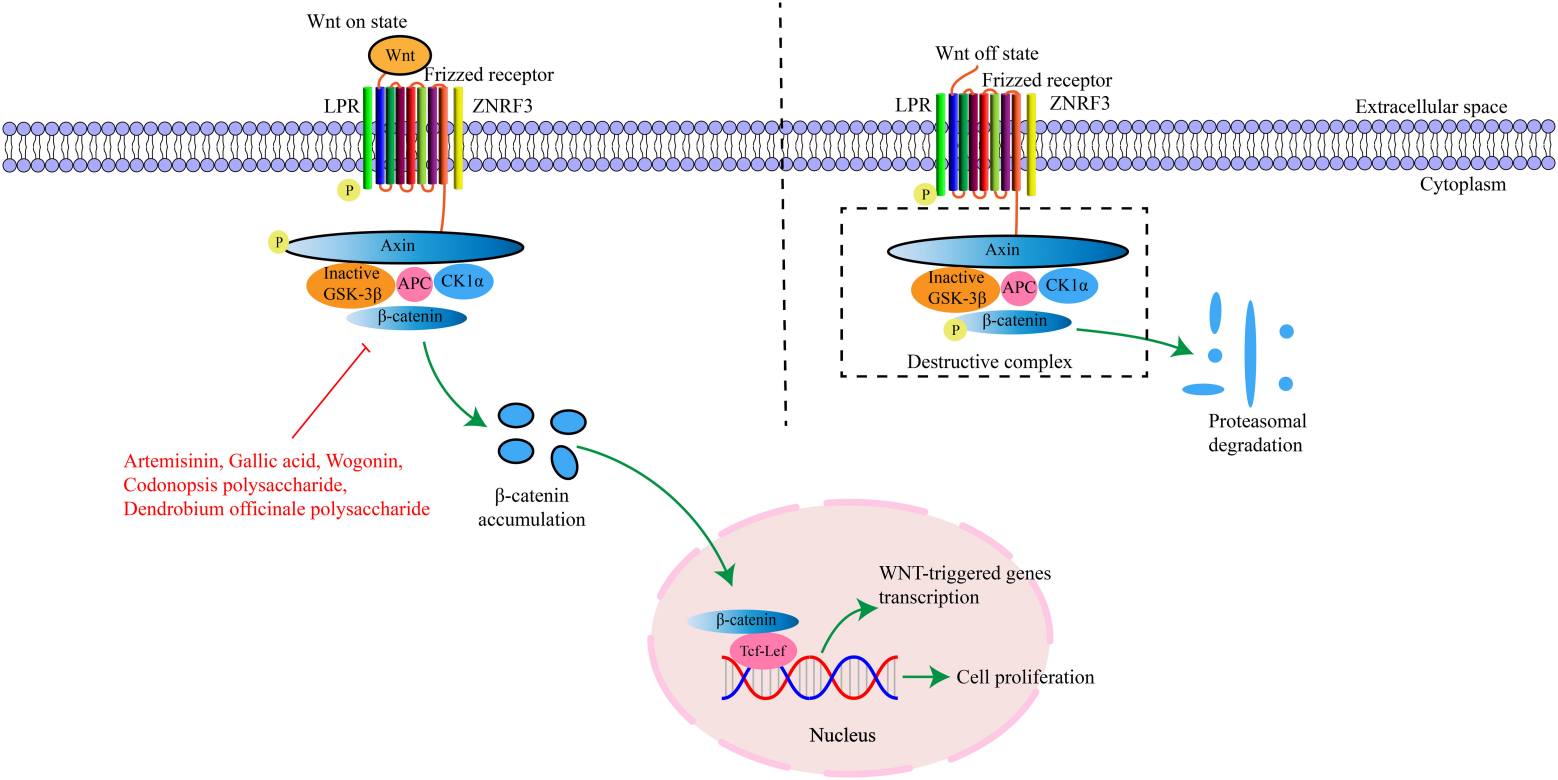
The interaction of key factors upstream and downstream in the Wnt/β-catenin signaling pathway.

Wnt proteins belong to a secreted glycoprotein family and are conserved in all metazoans. A total of 19 different genes encoding Wnt ligands have been identified from the human and mouse genomes (85). These ligands are cysteine-rich proteins with approximately 350–400 amino acids and contain an N-terminal signal peptide for secretion (86). β-catenin is a multifunctional protein that plays an important role in adherens junctions and Wnt signaling (87). In the absence of Wnt signals, the cytoplasmic level of β-catenin remains low, and when the Wnt signal is activated, β-catenin is stabilized and accumulated (88, 89). The β-catenin protein has 781 amino acid residues in humans and consists of a central region (residues 141-664) composed of 12 imperfect armadillo repeat sequences (R1-12) flanked by different amino-terminal domains and carboxyl-terminal domains (90). The Wnt/β-catenin pathway comprises four segments-membrane, cytoplasmic, nuclear, and extracellular signal (91), and involves genes and proteins such as Wnt1, Wnt3a, Wnt5a, glycogen synthase kinase 3β, cellular myelocytomatosis (c-Myc), and matrix metallopeptidase (92). This signaling pathway can regulate cell proliferation, differentiation, and apoptosis, and is critical for maintaining cellular homeostasis (86). Genetic and epigenetic alterations of the Wnt/β-catenin pathway can induce abnormal activation and contribute to cancer development. It has been found that continuous activation of the Wnt/β-catenin pathway in cancer is closely related to various oncogenic processes, including increased cell proliferation and EMT, as well as the maintenance of the self-renewal ability of cancer stem cells (93). The key targets in the Wnt/β-catenin signaling pathway and their upstream and downstream relationships are depicted in **Figure 3**.

### 4.2 Wnt/β-catenin pathway and PLGC

The Wnt/β-catenin signaling pathway is a key pathway regulating PLGC and inducing occurrence and metastasis of PLGC and GC, and its abnormal activation is closely related to PLGC occurrence and development (94). Studies found HP infection can promote the activation of Wnt/β-catenin signaling pathway in mice, accelerate the proliferation of gastric mucosal epithelial cells, inhibit apoptosis, and gradually appear abnormal cell proliferation and differentiation, triggering PLGC such as gastric mucosal atrophy, intestinal metaplasia and dysplasia (95). Moreover, Moluodan improved gastric tissue damage and prevented/reversed PLGC occurrence and progression by inhibiting Wnt/β-catenin signaling pathway activation (96). In PLGC state, gastrin-17, epithelial growth factor, and other molecules levels increase, activating Wnt signaling pathway. β-catenin accumulates in nucleus, elevating VEGF expression and tumor microvascular formation. β-catenin also stimulates active expression of downstream genes c-Myc and Cyclin D1. After obtaining nutritional support from microvessels, cells enter an irregular state of proliferation and division, infiltrate and metastasize around, and finally lead to gastric mucosal carcinogenesis, manifested as EMT or accompanied by IM and Dys appearance (97, 98). Mucin 5AC (MUC5AC) and Mucin 6 (MUC6) play important protective roles on gastric mucosal epithelium, with high expression in normal gastric mucosa and low expression in PLGC (99). Wang P et al. (100) found that inhibiting the Wnt/β-catenin signaling pathway and Wnt signaling pathway promoter Wnt3a can significantly improve MUC5AC and MUC6 expression and inhibit PLGC occurrence and development. Meanwhile, abnormal Wnt/β-catenin signaling pathway activation significantly affects gastric mucosal tissue cell division and carcinogenesis. Inhibiting this pathway reduces gastric mucosal damage and inflammation and delays PLGC progression (101). In addition, abnormal activation of the Wnt/β-catenin pathway promotes the expression of cell cycle-related proteins, which accelerates the process of cell cycle and enhances the ability of cell proliferation. This excessive proliferation can lead to an increase in the number and layer of gastric mucosal epithelial cells, gradually breaking the balance between cell proliferation and apoptosis, laying the foundation for the further development of PLGC (93, 102).

### 4.3 Effective components of traditional Chinese medicine regulate Wnt/β-catenin signaling pathway to treat PLGC

Artemisinin is a component of traditional Chinese medicine extracted from Artemisia annua with anti-inflammatory, anti-tumor, anti-fungal and immunomodulatory functions. Studies have found that Artemisinin can effectively inhibit the proliferation, invasion and migration of GC cells and its mechanism of action may be related to inhibiting the activation of the Wnt/β-catenin signaling pathway (27). Codonopsis polysaccharide is mainly extracted from the traditional Chinese medicine Codonopsis, with immunomodulatory and anti-tumor functions. Existing studies have confirmed that Codonopsis polysaccharide can inhibit the Wnt/β-catenin signaling pathway, affect the transcriptional activation of downstream target genes such as c-Myc and Cyclin D1, and promote the down-regulation of gene expression closely related to cell proliferation, thereby inhibiting the excessive proliferation of gastric mucosal cells, inducing cell differentiation in the normal direction, promoting apoptosis, and reversing the development of PLGC (28). Gallic acid is a natural component extracted from traditional Chinese medicinal materials such as gallnut and rhubarb with anti-inflammatory, antioxidant, antiviral and antibacterial functions. Liao W et al. (29) found that Gallic acid can down-regulate the Wnt/β-catenin signaling pathway, inhibit the EMT process, prevent the malignant behavior of PLGC cell proliferation and induce arrest at the G0/G1 phase, thereby blocking or delaying progression of PLGC to GC. Wogonin is mainly extracted from the root of Scutellaria baicalensis, with anti-inflammatory, antibacterial and anti-tumor functions. Studies have shown that Wogonin has the ability to inhibit the proliferation, migration and invasion of GC cells and can induce apoptosis and its mechanism of action may be related to inhibiting activation of the Wnt/β-catenin signaling pathway (30). Dendrobium officinale Polysaccharide mainly exist in the stems of Dendrobium officinale, with antioxidant, anti-tumor and immunomodulatory functions. Zhao Y et al. (94) found that Dendrobium officinale Polysaccharide can inhibit MNNG-induced PLGC by regulating the Wnt/β-catenin pathway and changing endogenous metabolites. In summary, traditional Chinese medicine active ingredients have demonstrated good efficacy in regulating the Wnt/β-catenin signaling pathway to treat PLGC. Through regulation of the Wnt/β-catenin signaling pathway, the state of gastric mucosa can be effectively improved, the proliferation ability of PLGC cells can be reduced, and the normal growth and differentiation of cells can be promoted, thus achieving the purpose of treating PLGC.

## 5 PI3K/AKT signaling pathway

### 5.1 Structure and biological function of PI3K/AKT pathway

**Figure 4:**
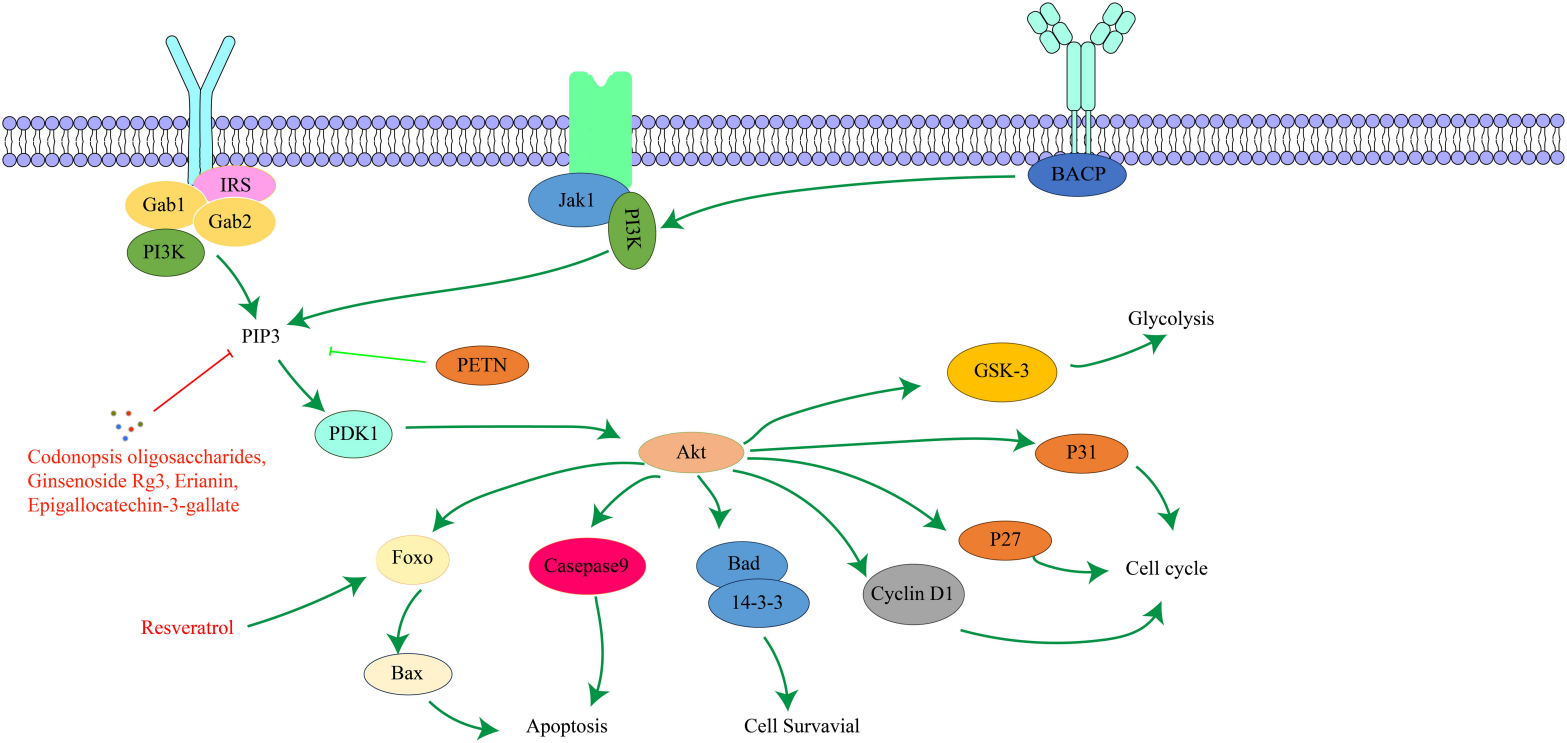
The interaction of key factors upstream and downstream in the PI3K/AKT signaling pathway.

PI3K is a family of lipid kinases that convert phosphatidylinositol to phosphatidylinositol trisphosphate (103). Based on structure, activation mode, and substrate specificity, it is divided into class I, class II, and class III (104). AKT is a serine/threonine protein kinase, and its active region shares high sequence similarity with protein kinase A and protein kinase C (105). There are three AKT isoforms, AKT-α, AKT-β, and AKT-γ (106). AKT has three main domains: the PH domain, which binds phospholipids and mediates cytoplasmic translocation of activated AKT; the catalytic domain, which has serine/threonine kinase and phosphatidylinositol kinase activity and catalyzes specific biochemical reactions; and the regulatory domain, which contains major phosphorylation sites, such as Ser473 (107). The PI3K/AKT signaling pathway is an important apoptotic pathway and plays a crucial role in cell survival. It regulates fundamental functions like cell proliferation, growth, survival, and metabolism and ensures appropriate cellular responses to external stimuli or damage (108–110). The PI3K/AKT signaling pathway is an important tumor development signaling route. Abnormal activation of this pathway is closely related to tumor cell invasion, metastasis, EMT, the immune microenvironment, and drug resistance of cancer cells, and it is one of the important mechanisms of tumorigenesis and development (111). The key targets in the PI3K/AKT signaling pathway and their upstream and downstream relationships are depicted in **Figure 4**.

### 5.2 PI3K/AKT pathway and PLGC

Recent years have seen growing recognition of the role of the PI3K/AKT signaling pathway in GC (112, 113). HP is a major pathogenic factor leading to chronic gastritis and GC. Studies have found that HP infection can recruit and activate PI3K, which in turn activates the PI3K/AKT signaling pathway. This abnormal activation can cause excessive proliferation of gastric mucosal cells, resulting in thickening of gastric mucosal tissue, laying the foundation for the occurrence of PLGC (114). The PI3K/AKT signaling pathway is a key regulatory mechanism for cell proliferation and apoptosis. Under normal cell hypoxia conditions, this signaling pathway is inhibited and promotes cell apoptosis by regulating the activity of the Bcl-2 protein family, exacerbating cell damage. However, under PLGC hypoxia stimulation, the PI3K/AKT signaling pathway is abnormally activated, hindering normal cell apoptotic processes (115). Specifically, AKT activation can inhibit apoptosis by phosphorylating various apoptosis-related proteins, allowing infected cells to survive and increasing the risk of PLGC transforming into GC (116). Other studies have shown significantly increased PI3K/AKT expression levels in CAG, IM, and GC, indicating a positive correlation between PI3K/AKT expression and PLGC progression (117). Moreover, the PI3K/AKT signaling pathway is an important mechanism for autophagy regulation and is closely related to PLGC occurrence and development. Appropriate autophagy level restoration significantly improves PLGC pathological state (118).

### 5.3 Effective components of traditional Chinese medicine regulate PI3K/AKT signaling pathway in the treatment of PLGC

Resveratrol, which can be derived from the traditional Chinese medicine Polygonum cuspidatum, is a compound naturally presentoccurring compound in these plants andwith has various effects such including as anti-inflammatory, antibacterial, antioxidant, anti-tumor, and immune regulation. Studies have found that Resveratrol increases phosphorylation by regulating the PI3K/AKT signaling pathway, activates Forkhead box O4, and then inhibits the expression of Chenodeoxycholic acid-induced gastric mucosal intestinal metaplasia markers, preventing the further development of PLGC (32). Codonopsis oligosaccharides are mainly extracted from the roots of Codonopsis pilosula, and have various biological activities such as anti-inflammatory, regulating intestinal flora, and enhancing immunity. Studies have confirmed that Codonopsis oligosaccharides can improve the hypoxic injury of normal gastric mucosal cells and induce GC cell apoptosis. It can significantly improve the gastric mucosal injury of PLGC rats by inhibiting inflammatory response, antioxidant effect, regulating the balance between apoptosis and proliferation, and adjusting metabolic disorders. Its mechanism of action is closely related to inhibiting the abnormal activation of PI3K/AKT signaling pathway (33). Ginsenoside Rg3 is mainly extracted from the dried roots, stems and leaves, fruits and other parts of Panax panax, and can also be extracted from American ginseng, Panax notoginseng and other plants. It has significant anti-tumor effects. A study showed that ginsenoside Rg3 combined with chemotherapy can improve the clinical efficacy of digestive system cancer and reduce the side effects of digestive system cancer caused by treatment (119), The effectiveness of this combination treatment method provides a basis for further research and development of ginsenoside Rg3 in the treatment of digestive system tumors. Liu W et al. (34) found that Ginsenoside Rg3 alleviated abnormal glycolysis in PLGC mice by down-regulating the expression of PI3K/AKT, lactate dehydrogenase A, hexokinase II and microRNA (miRNA) -21, indicating that Ginsenoside Rg3 can inhibit the proliferation of PLGC cells and induce apoptosis by down-regulating the PI3K/AKT signaling pathway. Epigallocatechin-3-gallate is an important active component in green tea and has a significant effect on cancer (120). Zhu F et al. (35) found that Epigallocatechin gallate improved the pathological state of gastric mucosa in PLGC rats and played a role in promoting apoptosis in PLGC rats. Its mechanism of action may be related to the inhibition of PI3K/AKT pathway. In addition, Erianin, as the main active ingredient of Dendrobium, also shows good clinical efficacy and medicinal value in preventing PLGC. Studies have found that Erianin can significantly reduce the protein expression levels of PI3K/AKT, Mouse double microgene 2, Cyclin D1 and phospho-glycogen synthase kinase 3beta, and increase the protein expression level of p21, suggesting that Erianin can treat PLGC by regulating the Harvey rat sarcoma viral oncogene homolog-PI3K-AKT signaling pathway (36).

In conclusion, the active ingredients of traditional Chinese medicine have demonstrated good efficacy and potential in the treatment of PLGC by regulating the PI3K/AKT signaling pathway. Components such as Resveratrol, Codonopsis oligosaccharides, Ginsenoside Rg3, Epigallocatechin-3-gallate, and Erianin inhibit PLGC proliferation and induce apoptosis through multiple mechanisms, while also showing regulatory effects on inflammation, providing a new direction for early GC intervention.

## 6 Sonic Hedgehog signaling pathway

### 6.1 The structure and biological function of Sonic Hedgehog pathway

**Figure 5:**
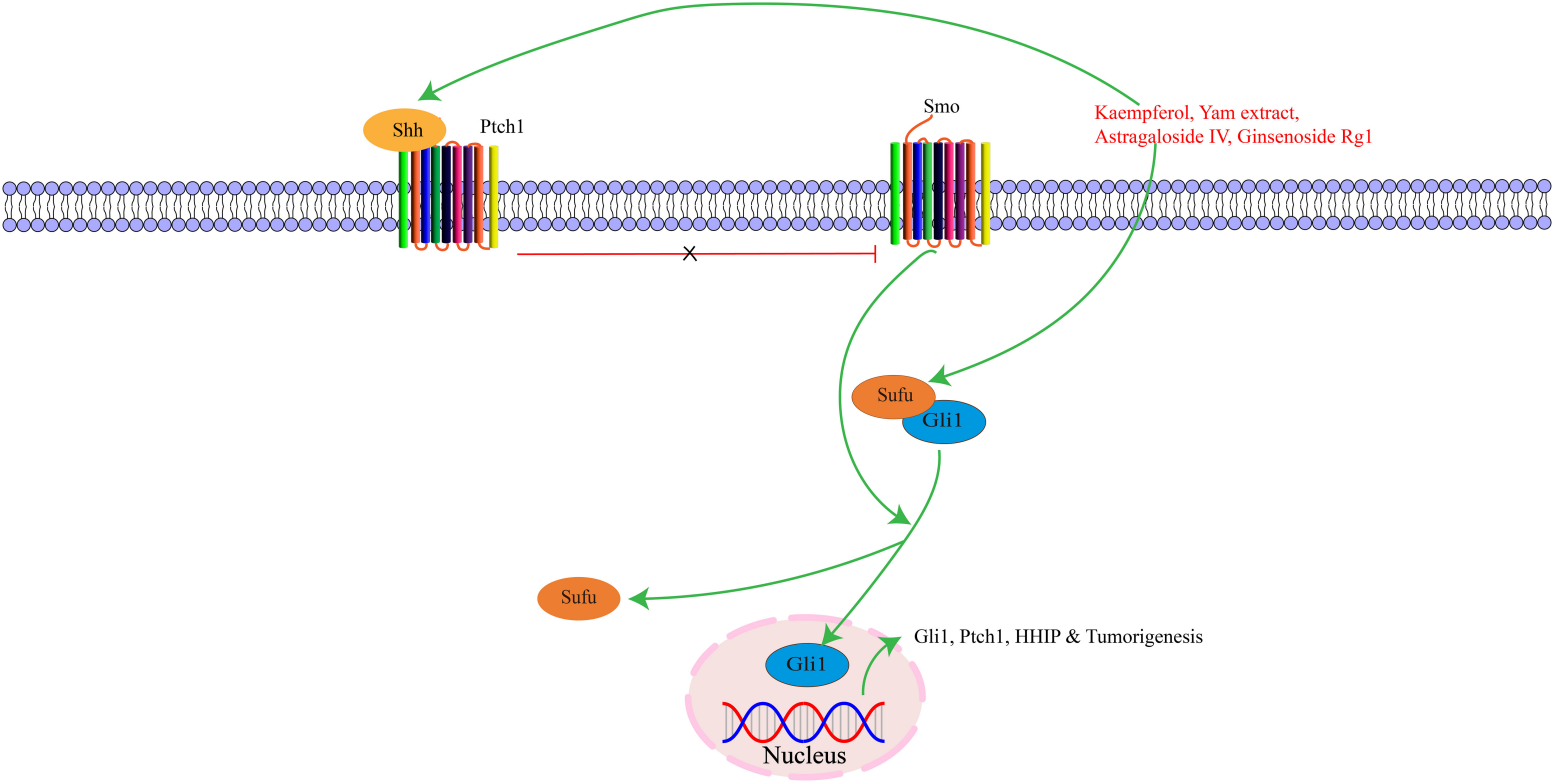
The interaction of key factors upstream and downstream in the Shh signaling pathway.

Activation of the Shh pathway begins with exogenous ligands binding to Hedgehog proteins. There are three major types: Shh, Indian Hedgehog (Ihh), and Desert Hedgehog (Dhh). Among these, Shh is the most critical component, initiating signals and regulating cell growth and differentiation (121). Shh signaling pathways consist of Hedgehog ligands, two transmembrane protein receptors [Ptch and Smoothened (Smo)], and the downstream transcription factor glioma-associated oncogene (Gli) protein. During embryonic development, this pathway is essential for the growth and patterning of various tissues, and also maintains adult tissue homeostasis (122). Hedgehog signaling pathway activation promotes cell proliferation via cell cycle regulation. Shh regulates gastric mucosal growth and differentiation through autocrine mechanisms and forkhead box L1-mediated epithelial-mesenchymal interactions (123). The Hedgehog signaling pathway plays a critical role in the growth and regeneration of cell tissues, regulating cell proliferation and differentiation to maintain proper tissue morphology and function (124, 125). Moreover, the Shh signaling pathway can participate in various oncogenic stages of different tumors, and abnormal activation of this pathway can promote tumor cell growth, proliferation, and invasion, accelerating tumor progression (126). The key targets in the Shh signaling pathway, as well as its upstream and downstream relationships, are illustrated in **Figure 5**.

### 6.2 Sonic Hedgehog pathway and PLGC

Studies have found that Shh, Smo, Gli1, Cyclin D1 and Cyclin E1 are highly expressed in PLGC rat tissues. Abnormal conduction of the Shh pathway leads to impaired differentiation of gastric mucosal cells, decreased gastric acid secretion, and accelerated transformation of gastric cells to a precancerous state (127). Shh pathway can affect the inflammatory microenvironment of gastric mucosa by regulating the secretion of inflammatory factors and the infiltration of inflammatory cells. Activation of Shh pathway can induce gastric mucosal cells to secrete inflammatory factors, attract inflammatory cells to aggregate, and form a chronic inflammatory environment. Continuous inflammatory stimulation can lead to gastric mucosal cell damage and repair imbalance, promoting the progress of PLGC (128). Gastric mucosal atrophy and dysplasia were associated with increased expression of Shh, Smo, Gli1, and Cyclin D1 proteins, and reduced expression of Ptch1, confirming involvement of the Shh signaling pathway in PLGC occurrence and development (129). The Hedgehog signaling pathway is critical for gastritis-to-PLGC transformation. Bacterial infection triggers Hedgehog-mediated conversion of the gastric mucosa from a pro-inflammatory to a precancerous state. Gastric mucosal inflammation and related inflammation can induce parietal cell atrophy and further develop into intestinal metaplasia through the Hedgehog pathway (130). The Shh pathway is highly expressed in parietal cells, and further reinforces the connection with PLGC by regulating the proliferation and differentiation of stem cells, the maturation and differentiation of gastric epithelial cells, and the secretion of gastric acid by parietal cells (131). In addition, upregulation of the Shh signaling pathway is associated with enhanced proliferation of tumor cells. Abnormal activation of the Shh signaling pathway promotes the proliferation and survival of PLGC cells, while inhibiting normal apoptosis of cells, leading to accumulation of PLGC cells and increasing the risk of PLGC-to-GC transformation (132).

### 6.3 Effective components of traditional Chinese medicine regulate Sonic Hedgehog signaling pathway to treat PLGC

Kaempferol is a flavonoid compound widely found in vegetables, Chinese herbal medicine, and other natural plants. It can be extracted from Chinese medicine Bupleurum, Licorice, and Eucommia ulmoides. It has various effects such as anti-cancer, anti-inflammatory, antioxidant, antibacterial, and antiviral. Tu W et al. (37) found that kaempferol can repair gastric mucosal damage, affect Hedgehog signaling pathway transduction, and reduce serum inflammatory factors IL-6 and IL-1β levels by regulating the Hedgehog signaling pathway, thus playing a role in CAG treatment. Yam extract can down-regulate serum gastrin expression and up-regulate serum epidermal growth factor and basic fibroblast growth factor receptor levels in gastric mucosa. Studies have found that yam extract can promote Shh downstream signaling pathway expression and activate its ligands. By regulating Ptch1, Smo, Gli1, and other protein expressions, it can improve gastric tissue transportation, gastric mucosal low acid environment, oxidative stress, and digestive enzyme secretion, thereby improving gastric mucosal pathological changes in PLGC rats (38). Astragaloside A is mainly extracted from the root of Astragalus, and ginsenoside Rg1 can be extracted from traditional Chinese medicine ginseng or Panax notoginseng. Both have anti-inflammatory, antioxidant, and anti-tumor functions. Studies have shown that Astragaloside IV and Ginsenoside Rg1 have certain activation effects on key factors of Hedgehog signaling pathway, and further maintain the dynamic balance of cell proliferation and apoptosis by regulating the expression of feedback factors in the signaling pathway, thereby improving the pathological state of gastric mucosa in CAG rats and inhibiting the progression of PLGC (39).

In conclusion, the effective ingredients of traditional Chinese medicine show unique advantages in the treatment of PLGC by regulating the Shh signaling pathway. The anti-PLGC mechanism of these active ingredients, such as Kaempferol, Yam extract, Astragaloside IV, and Ginsenoside Rg1, not only involves the regulation of the Shh signaling pathway but also includes various effects such as anti-inflammatory, antioxidant, and mucosal repair.

## 7 JAK/STAT signaling pathway

### 7.1 The structure and biological function of JAK/STAT pathway

**Figure 6:**
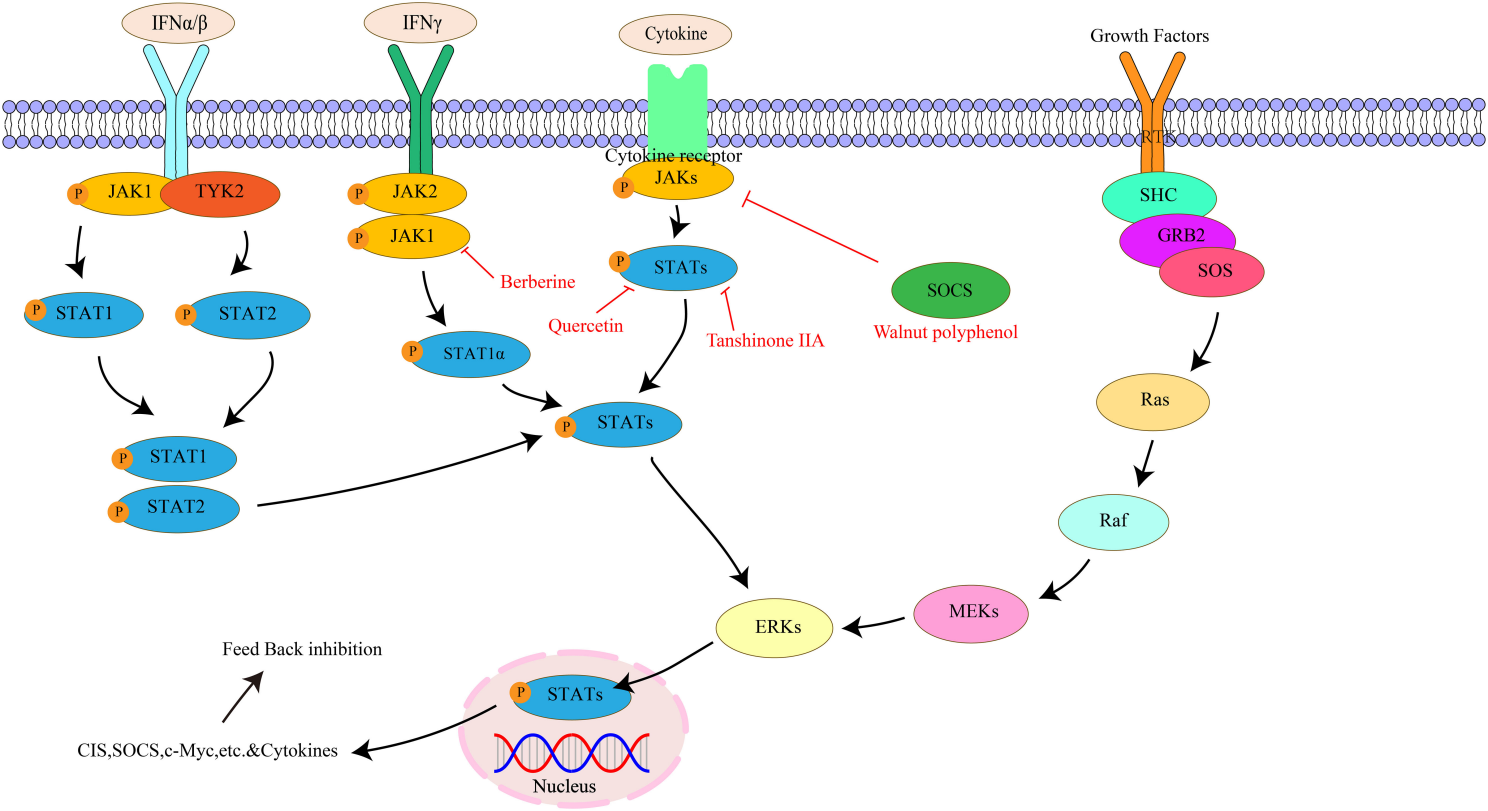
The interaction of key factors upstream and downstream in the JAK/STAT signaling pathway.

The JAK/STAT pathway is composed of three parts: cell receptor, JAK protein, and STAT protein (133). The JAK family belongs to the non-transmembrane tyrosine kinase family and mainly includes four family members: JAK1, JAK2, JAK3, and TYK2. JAK1 regulates cytokines, JAK2 mediates ligand and receptor binding, and JAK1, JAK2, and tyrosine-kinase 2 are ubiquitous in all tissues. In contrast, JAK3 is only present in bone marrow, lymphatic system, endothelial cells, and vascular smooth muscle cells (134, 135). STATs belong to potential cytoplasmic transcription factors, including seven subtypes of STAT1, STAT2, STAT3, STAT4, STAT5A, STAT5B, and STAT6 (136). The JAK/STAT pathway can promote the proliferation of various cell types, including stem cells and adult cells. It regulates cell proliferation by upregulating genes related to the cell cycle (137). Meanwhile, the JAK/STAT signaling pathway also plays an important role in immunosuppression, stem cells, and pre-metastatic niches (138). Studies have found the JAK/STAT pathway can participate in the generation and differentiation of adipocytes, affecting the body’s energy metabolism and fat storage (139). Most importantly, the JAK/STAT pathway plays a key role in gastrointestinal tumors and inflammatory diseases. Activation of this pathway maintains elevated expression of inflammatory cells and factors, promoting further disease development (140). The key targets in the JAK/STAT signaling pathway, as well as its upstream and downstream relationships, are illustrated in **Figure 6**.

### 7.2 JAK/STAT pathway and PLGC

Wang S et al. (141) found that bile reflux can promote the occurrence of PLGC by activating the IL-6/JAK/STAT signaling pathway, and STAT3 inhibition can reduce this carcinogenic effect. HP infection induces many pro-inflammatory signaling pathways and is a major risk factor for GC development. Li X et al. (142) found that STAT1 is activated in HP-positive gastritis and is significantly elevated in GC, with its target gene programmed cell death-ligand 1 (PD-L1), confirming that HP-induced activation of STAT1 and PD-L1 expression may prevent immune surveillance in the gastric mucosa, promoting PLGC to GC development. The JAK/STAT cascade is the primary signal transduction pathway in cytokine and growth factor signaling, regulating diverse cellular processes including cell proliferation, differentiation, migration, and survival. Abnormal expression of JAK/STAT not only promotes cancer cell proliferation and survival but also participates in PLGC formation (143). When the transcription factor STAT3 is cytokine-activated, the JAK/STAT signaling pathway is involved in the transcription of genes implicated in cell proliferation, apoptosis, and other cellular processes, acting as an oncogene to enhance metastatic potential and tumorigenesis (144). Moreover, the JAK/STAT pathway is also related to changes in the tumor microenvironment. It can induce the expression of various inflammatory factors, forming a chronic inflammatory microenvironment. Long-term inflammatory stimulation will continue to damage gastric mucosal cells. At the same time, various cytokines and reactive oxygen species in the inflammatory microenvironment can induce genetic mutations in gastric mucosal cells and promote the development of PLGC (145).

### 7.3 Effective components of traditional Chinese medicine regulate JAK/STAT signaling pathway in the treatment of PLGC

Walnut has medicinal value in nourishing the kidneys and lungs, moistening the intestines, and promoting bowel movements. Walnut polyphenols are extracts from Walnuts with anti-inflammatory, antioxidant, and immune-enhancing functions. Park JM et al. (40) found that Walnut polyphenol extracts can inhibit STAT3 activation by increasing suppressor of cytokine signaling-1 expression, thereby preventing the release of inflammatory cytokines IL-6 and TNF-α and inhibiting HP-induced CAG. Quercetin exists in vegetables, fruits, cereals and other foods as well as a variety of medicinal plants, with various pharmacological effects. Studies have found that Quercetin can inhibit the JAK/STAT pathway and reduce inflammation and cell proliferation by inhibiting cytokine release, including IL-6 and TNF-α (41). Berberine, extracted from traditional Chinese medicines Coptis chinensis and Phellodendron chinensis, exhibits anti-inflammatory and antibacterial effects. Yang T et al. (42) demonstrated that Berberine can regulate macrophage polarization via the IL-4/STAT6 signaling pathway, reducing gastric mucosal inflammation induced by HP infection and effectively treating HP-induced CAG by inhibiting the interferon regulatory factor 8-interferon-gamma signaling axis, thereby preventing further progression to PLGC. Tanshinone IIA is mainly extracted from the root of Salvia miltiorrhiza, exhibits strong anti-inflammatory and antioxidant capacity (146). Tanshinone IIA has been found to possess significant anti-proliferative and pro-apoptotic effects on GC cells, inducing apoptosis and inhibiting STAT3 phosphorylation at the Tyr705 residue, thereby exerting its anti-proliferative effects in PLGC lines through inhibition of STAT3 activation (43).

In conclusion, the effective ingredients of traditional Chinese medicine show potential to treat PLGC by regulating the JAK/STAT signaling pathway. Walnut polyphenol, Quercetin, Berberine, Tanshinone IIA, and other active ingredients of traditional Chinese medicine inhibit PLGC cell proliferation and migration through various mechanisms, reduce inflammation, and protect against gastric mucosal damage, thereby inhibiting PLGC progression.

## 8 MAPK pathway

### 8.1 The structure and biological function of MAPK pathway

**Figure 7:**
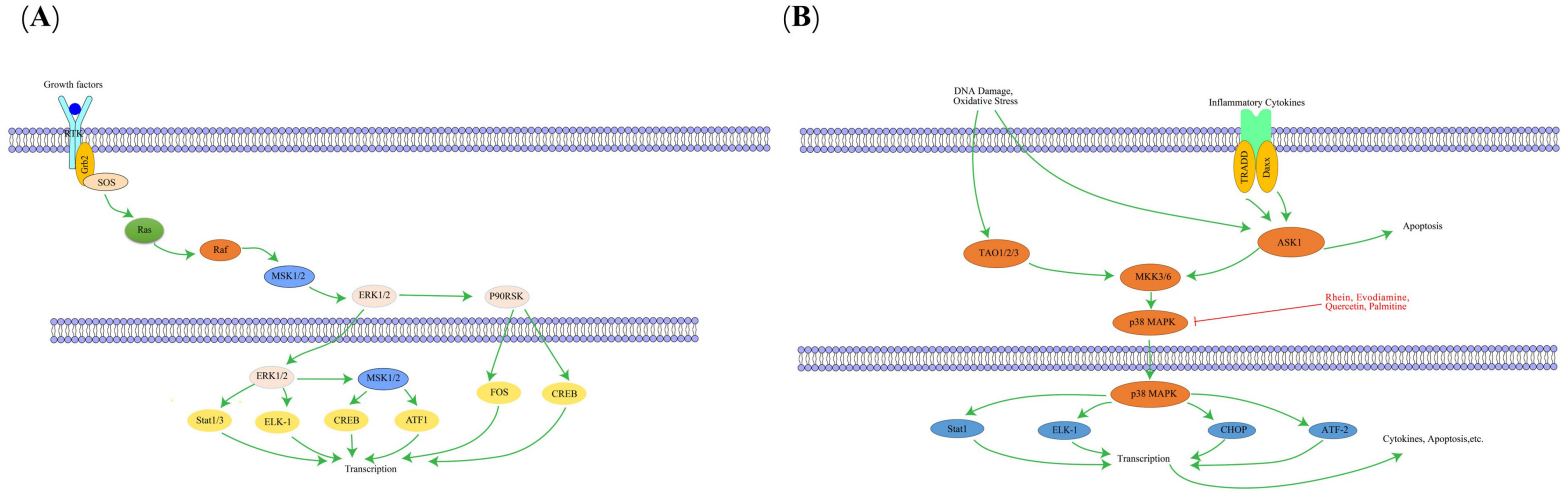
The interaction of key factors upstream **(A)** and downstream **(B)** in the MAPK signaling pathway.

The MAPK pathway is a highly conserved tertiary kinase cascade system, composed primarily of three key kinases: MAPK kinase (MAP2K), MAPK kinase kinase (MAP3K), and MAPK itself (147). MAPK signaling initiates with MAPKKK, which phosphorylates MAPKK; subsequently, MAPKK phosphorylates MAPK, ultimately activating it (148). The MAPK pathway subfamily includes the extracellular signal-regulated kinase pathway, c-Jun N-terminal kinase (JNK) pathway, big mitogen-activated protein kinase 1 pathway, and p38 pathway, with the p38 pathway being particularly important in regulating inflammation and transmitting signals to various physiological stress pathways (149, 150). MAPK is primarily located in the cytoplasm and mediates diverse cellular responses by transducing extracellular signals, regulating cell growth and apoptosis (151). Studies reveal the MAPK pathway promotes aggregation and activation of white blood cells, crucial for regulating inflammatory responses (152). Moreover, the MAPK pathway plays crucial roles in cell proliferation and differentiation; abnormal activation can lead to abnormal cell proliferation and malignant transformation (153). The key targets in the MAPK signaling pathway, as well as its upstream and downstream relationships, are illustrated in **Figure 7**.

### 8.2 MAPK pathway and PLGC

MAPK pathway components may be dysregulated during PLGC occurrence, leading to overactivated cell proliferation and inhibited apoptosis, providing a basis for PLGC occurrence. Studies have shown that the activation of the MAPK pathway is closely related to the occurrence of PLGC. When gastric mucosal cells are affected by carcinogenic factors, they will activate related kinases in the MAPK pathway, thereby phosphorylating and activating a series of transcription factors. These transcription factors can bind to the promoter region of cyclin genes and promote the expression of cyclin, resulting in abnormal proliferation of gastric mucosal cells. This is an important feature of PLGC (154, 155). During the IM process, abnormal growth factor stimulation or inflammatory factors may lead to abnormal activation of the MAPK pathway. Continued activation of this pathway results in abnormal cell cycle regulation, accelerating malignant transformation of cells, and promoting the development of PLGC (156). At the same time, HP infection induces oxidative stress in gastric mucosal cells, thereby activating the MAPK pathway. Activated MAPK pathway promotes cell proliferation and inhibits apoptosis, allowing damaged cells to survive and proliferate, increasing the risk of PLGC (157). Furthermore, MAPK pathway also interacts closely with inflammatory factors in PLGC, which can activate MAPK pathway, and activation of MAPK pathway can further regulate the expression of inflammation-related factors and promote the continuation of inflammatory response. Long-term chronic inflammation can lead to repeated damage and repair of gastric mucosal tissue, increasing the possibility of PLGC (158, 159).

### 8.3 Effective components of traditional Chinese medicine regulate MAPK signaling pathway in the treatment of PLGC

Rhein, an active ingredient found in various traditional Chinese medicines (e.g., rhubarb, aloe vera), possesses anti-inflammatory and anti-tumor properties. Liu S et al. (44) discovered that rhubaric acid can reverse phosphorylation of JNK and p38 in the gastric mucosa of CAG mice induced by HP infection, inhibit expression of inflammatory factors TNF-α, IL-1β, and IL-6, suggesting that rhubaric acid reduces gastric mucosal damage in mice by inhibiting the MAPK signaling pathway and reducing inflammation and oxidative stress. Evodiamine is mainly extracted from the fruit of traditional Chinese medicine Evodia rutaecarpa, also possesses anti-inflammatory and anti-tumor activities. Yang JY et al. (45) confirmed that Evodinine can inhibit the activation of MAPK pathway induced by HP infection, thereby inhibiting the release of inflammatory mediators by inflammatory cells, reducing the inflammatory damage of gastric mucosa, and slowing down the development of PLGC. Studies have found that the main components of reed root and turmeric drug pairs include Quercetin, palmitine, etc., and these traditional Chinese medicine active ingredients can effectively improve gastrointestinal function by regulating the MAPK signaling pathway and inflammatory factor target genes in CAG rats, relieving inflammation, repairing mucosal damage, and avoiding further CAG development (46).

In conclusion, the active ingredients of traditional Chinese medicine can effectively regulate the progression of PLGC by modulating the MAPK signaling pathway. Rhein, Evodiamine, Quercetin, Palmitine and other active ingredients of traditional Chinese medicine play important roles in this process.

## 9 Nrf2 signaling pathway

### 9.1 The structure and biological function of Nrf2pathway

**Figure 8:**
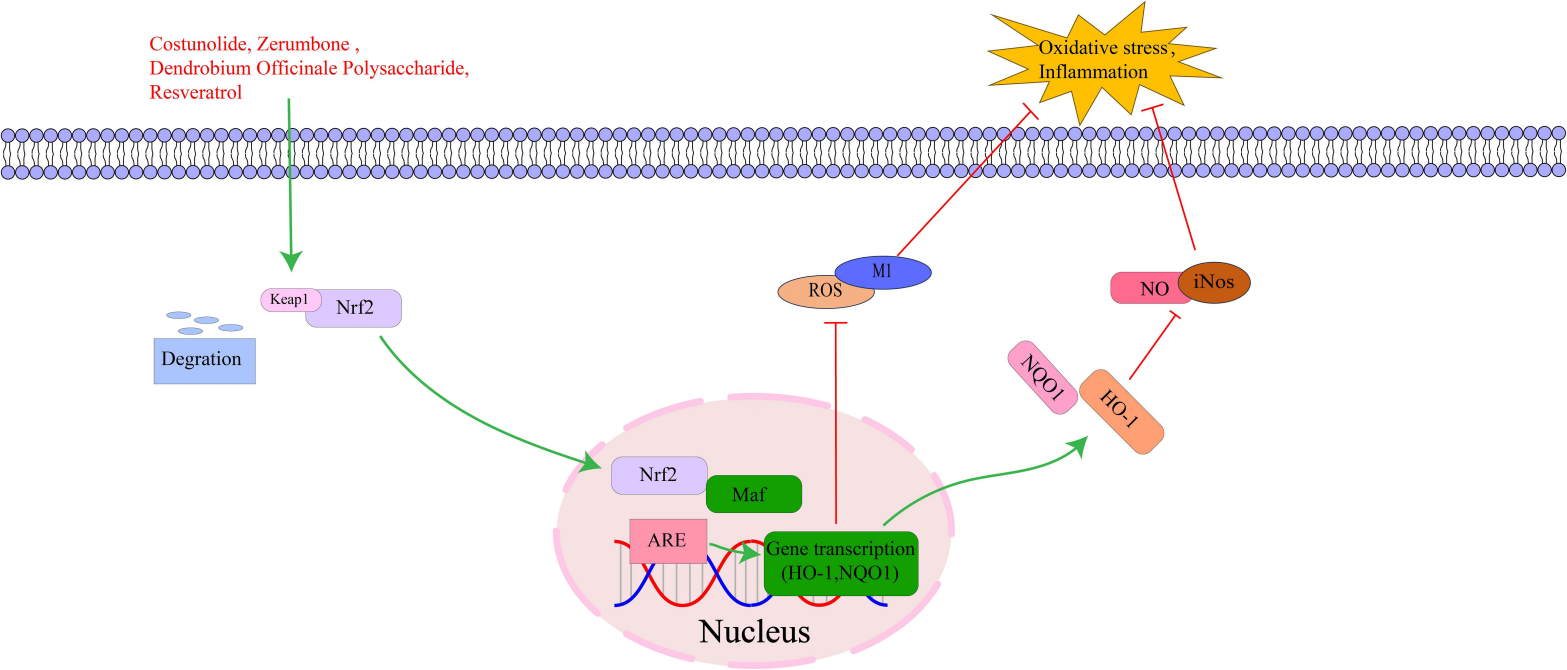
The interaction of key factors upstream and downstream in the Nrf2 signaling pathway.

Nrf2 is a basic leucine zipper (bZIP) transcription factor with multiple functional domains, primarily including Neh1-Neh7 (160). The Neh1 domain contains a bZIP that recognizes and binds to DNA sequences and is essential for Nrf2 to bind to antioxidant response elements in the promoter region of target genes (161). The Neh2 domain is primarily responsible for interacting with Keap1 protein. This domain contains two binding checkpoints, one is the ETGE motif and the other is the DLG motif, which play a key role in regulating the activity and stability of Nrf2 (162). Nrf2 can regulate the expression of antioxidant enzymes including superoxide dismutase, glutathione peroxidase, and catalase. These enzymes clear reactive oxygen species and active nitrogen in cells, maintain intracellular redox balance, and protect cells from oxidative damage (163). In addition, Nrf2 can not only indirectly affect the inflammatory response by regulating antioxidant enzyme production, but also directly regulate expression of some inflammation-related genes, thereby maintaining body inflammatory homeostasis and preventing excessive inflammation from causing tissue damage (164, 165). The key targets in the Nrf2 signaling pathway, as well as its upstream and downstream relationships, are illustrated in **Figure 8**.

### 9.2 Nrf2 pathway and PLGC

Abnormal activation of the Nrf2 pathway promotes proliferation of PLGC cells. Nrf2 can regulate expression of genes related to cell differentiation. In PLGC, this regulation may be disrupted, leading to failure of gastric mucosal epithelial cells to differentiate normally and appearance of precancerous lesions such as IM (166). The Nrf2 pathway plays a critical role in protecting the gastrointestinal tract from oxidative stress and chemical damage to the gastrointestinal mucosa. PLGC is often accompanied by an increase in oxidative stress, and activation of Nrf2 is a key mechanism for cells to cope with this stress. Activation of Nrf2 can upregulate antioxidant enzyme activity, which can reduce inflammation and reduce oxidative damage in cells, thereby slowing the progression of PLGC (167, 168). The Nrf2 pathway is also involved in regulating the balance of proliferation and apoptosis of gastric mucosal cells. Under normal conditions, the proliferation and apoptosis of gastric mucosal cells are in a state of dynamic balance. In PLGC, this balance is disrupted, and Nrf2 can restore it by modulating the expression of cyclin and apoptosis-associated proteins (169, 170).

### 9.3 Effective components of traditional Chinese medicine regulate Nrf2 signaling pathway to treat PLGC

Costunolide is mainly extracted from the roots of Aucklandia lappa, with exhibits various biological activities including anti-inflammatory, antioxidant, and anti-tumor effects. Studies have found that xylophane can promote the expression of Nrf2 in gastric tissue, initiate the transcription of a series of antioxidant enzyme genes, which can remove excessive reactive oxygen species and free radicals in cells, reduce the damage of oxidative stress to gastric tissue cells, thereby maintaining the normal physiological function of cells and preventing the development of gastric mucosa toward precancerous lesions (47). Zerumbone, which can be extracted from red ball ginger, possesses anti-inflammatory, antibacterial, and anti-tumor activities. Li L et al. (48) found that Zerumbone can up-regulate the expression of Nrf2 and heme oxygenase-1 (HO-1) proteins in gastric mucosa tissue, increase catalase and superoxide dismutase activities, increase glutathione levels, and reduce malondialdehyde levels, thereby reducing inflammation and exerting protective effects on gastric mucosa. Dendrobium Officinale Polysaccharide is the main active ingredient of Dendrobium officinale, with various effects such as antioxidant, anti-tumor, and immune regulation. Zhao Y. et al (171) found that Dendrobium Officinale Polysaccharide can promote Nrf2 expression in the gastric mucosa, enhance antioxidant activity, protect gastric mucosal cells from oxidative damage, reduce IM and Dys severity, and inhibit PLGC development. Additionally, Resveratrol, a traditional Chinese medicine extract, can increase Nrf2 and HO-1 expression levels in the gastric mucosa of HP infection, thereby exerting significant antioxidant and anti-inflammatory effects, reducing inflammatory damage to the gastric mucosa, and preventing PLGC progression (49).

In conclusion, the active ingredients of traditional Chinese medicine show significant potential in the treatment of PLGC by regulating the Nrf2 pathway. These active ingredients can activate the Nrf2 pathway to enhance antioxidant capacity, reduce oxidative stress damage to the gastric mucosa, and inhibit inflammation, regulate cell proliferation and apoptosis, and intervene in the occurrence and development of PLGC from multiple links.

## 10 Molecular markers and metastases immunotherapy

With the in-depth investigation of the mechanisms underlying the actions of active ingredients in traditional Chinese medicine, the significance of molecular marker research has gradually emerged. Molecular markers are bioactive substances—including genes, proteins, and metabolites—that are produced by tumor cells or altered by the host during tumor development. These markers can be employed in various applications, such as early diagnosis, prognostic evaluation, therapeutic target selection, and efficacy monitoring in tumors, playing a key role in guiding tumor immunotherapy. CfDNA is a free DNA fragment extracted from blood. Studies have demonstrated that the concentration of cfDNA is closely associated with the progression and staging of gastrointestinal tumors, and its variation may serve as a reliable biomarker for detecting early-stage GC (172, 173). In recent years, miRNAs, including miR-200a, miR-1236, and miR-559, have been extensively studied in the context of gastrointestinal tumor treatment. These miRNAs play a unique role in the initiation, progression, and metastasis of gastrointestinal tumors, thereby providing novel therapeutic targets and strategies (174). Studies have found that jumonji domain containing 3 (JMJD3) may serve as an important epigenetic therapeutic target and/or prognostic predictor in GC, and that inhibiting JMJD3 might offer an effective therapeutic strategy for this cancer (175). Chen Y et al. (176) demonstrated that lncRNAs play a crucial role in the epigenetic regulation of cancer cells—primarily through mechanisms such as DNA methylation, chromatin modification, gene transcription, translational regulation, and miRNA sponging—which regulate the expression of oncogenes or tumor suppressor genes and, consequently, affect tumor cell proliferation, metastasis, and apoptosis. Zhao Y et al. (31) reported that m1A regulatory genes can influence the biological behavior of gastrointestinal cancer cells by modulating signaling pathways including PI3K/AKT/mTOR and ErbB. In the research and treatment of GC, traditional Chinese medicine and molecular markers are interconnected in various ways. Traditional Chinese medicine can exert anti-cancer effects by modulating molecular markers. For instance, curcumin inhibits the expression of cyclin D1 and reduces Ki67 expression through the NF-κB signaling pathway, thereby suppressing the proliferation of GC cells (177). Additionally, resveratrol has been shown to inhibit EMT by downregulating metastasis-associated lung adenocarcinoma transcript 1, which in turn reduces the migration and invasion of GC cells (178).

Overall, the study of molecular markers paves the way for the development of novel tumor immunotherapy drugs and fosters continuous innovation in tumor immunotherapy by identifying new immunotherapy targets. With the ongoing advancement of technologies such as gene sequencing and proteomics, it is anticipated that additional molecular markers associated with the individualized characteristics of GC will be identified. Traditional Chinese medicine offers the potential for precision treatment based on these specific molecular markers. By leveraging big data analytics, it is possible to match patients’ molecular profiles with the active components of traditional chinese medicine, thereby enabling the formulation of more targeted therapeutic strategies, improving treatment outcomes, and minimizing unnecessary side effects. Furthermore, in-depth research is needed to elucidate the mechanisms by which traditional chinese medicine regulates GC-related molecular markers and to identify key pharmacologically active components and their corresponding targets. Utilizing modern pharmaceutical technologies, novel anti-GC traditional chinese medicine drugs with high efficacy, low toxicity, and consistent quality can be developed. Concurrently, integrating research on traditional chinese medicine quality markers will help ensure drug quality and therapeutic efficacy at the source, ultimately supporting the global advancement and acceptance of innovative traditional chinese medicine therapies.

## 11 Summary and prospects

The pathological process of PLGC is complex, involving a variety of cellular changes and potential risk factors. Progression from PLGC to GC involves activation of various signaling pathways, crucial for transmitting cellular physiological activities. Abnormal regulation of many signaling pathways occurs during PLGC occurrence and development, leading to disorders in cell proliferation, apoptosis, differentiation, and cell-to-cell interactions. Recent years have seen significant progress in regulating relevant PLGC signaling pathways with active Chinese medicine ingredients. Numerous studies have demonstrated that the active ingredients of traditional Chinese medicine can modulate several key signaling pathways, including HIF-1α, NF-κB, Wnt/β-catenin, PI3K/Akt, Sonic Hedgehog, JAK/STAT, MAPK, and Nrf2, among others. By exerting multi-target synergistic effects, these compounds can more comprehensively and effectively impede the progression of lesions. Given the limitations of current treatments for precancerous lesions of GC, the modulation of signaling pathways by traditional Chinese medicine provides a novel therapeutic approach. For patients who cannot tolerate surgery or experience adverse reactions to Western medicine, traditional Chinese medicine-based treatment regimens offer good tolerance and efficacy, presenting alternative treatment options. Moreover, research on traditional Chinese medicine’s impact on signaling pathways facilitates the transition from the study of traditional Chinese medicine compound prescriptions to the development of modern, innovative traditional Chinese medicine-based drugs. By elucidating the signaling mechanisms of traditional Chinese medicine’s active ingredients, modern scientific technologies can be employed to enhance drug development efficiency and success rates, providing new models and insights for the research and development of innovative therapies.

HP can promote the progress of PLGC by activating a variety of signaling pathways. When studying the regulation of relevant signaling pathways by active ingredients of traditional Chinese medicine in the treatment of PLGC, HP infection cannot be ignored as a key factor. For PLGC patients with HP infection, traditional Chinese medicine combined with western medicine can be used. For example, the use of active ingredients of traditional Chinese medicine such as berberine, curcumin, ginsenoside Rg3 with anti-HP effects, combined with proton pump inhibitors and antibiotics, while eradicating HP, the active ingredients of traditional Chinese medicine can regulate relevant signaling pathways, reduce inflammation, and promote gastric mucosal repair.

At present, the active ingredients of traditional Chinese medicine have shown some potential in regulating relevant signaling pathways in the treatment of PLGC, but research in this field still faces many challenges. First, the active ingredients of traditional Chinese medicine are complex and diverse, and their functions often result from synergistic effects of multiple targets and pathways, making it difficult to accurately analyze the mechanism of individual active ingredients, determine dominant targets and signaling pathways, and understand their relationships. Second, the pharmacokinetic properties of active ingredients of traditional Chinese medicine *in vivo*, including absorption, distribution, metabolism, and excretion, are not fully understood, hindering the determination of optimal dosage, dosage form, and route of administration in drug development, and limiting the effectiveness and safety evaluation of clinical application. Furthermore, most studies are still in the cell and animal experiment stage, with small clinical studies and limited sample sizes. There is a lack of large-scale, multicenter, randomized controlled clinical trials to fully verify the efficacy and safety of active ingredients of traditional Chinese medicine in PLGC treatment. Additionally, the synergistic or antagonistic mechanisms between active ingredients of different traditional Chinese medicines need further exploration. Therefore, future research should focus on further elucidating the targets and molecular mechanisms of active ingredients in traditional Chinese medicine. It is essential to leverage multidisciplinary approaches, such as systems biology and bioinformatics, to precisely analyze the regulatory effects of these active ingredients on signaling pathways related to gastric precancerous lesions. This approach will provide a solid theoretical foundation for clinical translation. Simultaneously, we should conduct large-scale, multi-center, randomized controlled clinical trials to verify the efficacy and safety of active ingredients of traditional Chinese medicine in humans, formulate a scientific and reasonable clinical trial plan, and clearly include criteria, exclusion criteria, efficacy evaluation indicators, etc., to track patients with gastric precancerous lesions who use drugs containing active ingredients of relevant traditional Chinese medicine, and analyze the correlation between their therapeutic effects and changes in signaling pathways, so as to provide reliable evidence-based medical evidence for the wide application of traditional Chinese medicine in the clinical treatment of PLGC.

In conclusion, the active ingredients of traditional Chinese medicine regulate related signaling pathways, demonstrating great potential and broad prospects for the treatment of PLGC. With the deepening of research and the innovation of technology, it is expected to bring new breakthroughs in the prevention and treatment of PLGC in the future, improve the early prevention and treatment level of GC, and benefit a large number of patients.
